# TRAP-induced PAR1 expression with its mechanism during AMI in a rat model

**DOI:** 10.1186/s12872-023-03118-w

**Published:** 2023-02-21

**Authors:** Ani Wang, Xinyuan Gu, Chunyang Wang, Yanhui Li, Fuhong Deng, Jie Fang, Naxia Chen, Qifu Li, Lilong Tang

**Affiliations:** 1 Department of Cardiology, The Fifth Affiliated Hospital of Sun Yat-Sen University, #52 Meihua East Road, Zhuhai, Guangdong People’s Republic of China; 2 Division of Geriatics, The First Affiliated Hospital of Hainan Medical University, Haikou, People’s Republic of China; 3 Yuebei Hospital, Shaoguan, People’s Republic of China; 4 Division of Cardiology, Tongji Hospital Affiliated to Huazhong Technology University, Wuhan, People’s Republic of China; 5 Division of Cardiology, Xiangtan Central Hospital, Xiangtan, People’s Republic of China; 6 Department of Neurology, The First Affiliated Hospital of Hainan Medical University, Haikou, People’s Republic of China

**Keywords:** PAR1, Rab11, Cardiomyocytes, Hypoxia, Cardiac function, AMI

## Abstract

**Background and objective:**

Protease-activated receptor 1 (PAR1) is crucial in individuals with acute myocardial infarction (AMI). The continuous and prompt PAR1 activation mainly dependent on PAR1 trafficking is essential for the role of PAR1 during AMI in which cardiomyocytes are in hypoxia. However, the PAR1 trafficking in cardiomyocytes specially during the hypoxia is still unclear.

**Methods and result:**

A rat AMI model was created. PAR1 activation with thrombin-receptor activated peptide (TRAP) had a transient effect on cardiac function in normal rats but persistent improvement in rats with AMI. Cardiomyocytes from neonatal rats were cultured in a normal CO2 incubator and a hypoxic modular incubator chamber. The cells were then subjected to western blot for the total protein expression and staining with fluorescent reagent and antibody for PAR1 localization. No change in total PAR1 expression following TRAP stimulation was observed; however, it led to increased PAR1 expression in the early endosomes in normoxic cells and decreased expression in the early endosomes in hypoxic cells. Under hypoxic conditions, TRAP restored the PAR1 expression on both cell and endosomal surfaces within an hour by decreasing Rab11A (8.5-fold; 179.93 ± 9.82% of the normoxic control group, n = 5) and increasing Rab11B (15.5-fold) expression after 4 h of hypoxia. Similarly, Rab11A knockdown upregulated PAR1 expression under normoxia, and Rab11B knockdown downregulated PAR1 expression under both normoxic and hypoxic conditions. Cardiomyocytes knocked out of both Rab11A, and Rad11B lost the TRAP-induced PAR1 expression but still exhibited the early endosomal TRAP-induced PAR1 expression under hypoxia.

**Conclusions:**

TRAP-mediated activation of PAR1 in cardiomyocytes did not alter the total PAR1 expression under normoxic conditions. Instead, it triggers a redistribution of PAR1 levels under normoxic and hypoxic conditions. TRAP reverses the hypoxia-inhibited PAR1 expression in cardiomyocytes by downregulating Rab11A expression and upregulating Rab11B expression.

**Supplementary Information:**

The online version contains supplementary material available at 10.1186/s12872-023-03118-w.

## Introduction

The binding of thrombin and protease-activated receptor 1 (PAR1) results in PAR1 cleavage, exposing its NH_2_ terminal, which functions as a ‘tethered peptide ligand’, leading to receptor activation. Hirudin can compete with thrombin for binding to this receptor and inhibit thrombin-PAR1 interaction. Thrombin receptor activating peptide (TRAP), a synthetic peptide comprising 5–14 amino acids (SFLLRN), resembles the tethered ligand sequence of PAR and acts as an agonist for selective activation of the receptors [1]. The thrombin-induced receptor cleavage leads to endocytosis of the thrombin-receptor complex and lysosomal degradation. The replenishment of these proteolytically-cleaved thrombin receptors on cardiomyocytes is the key to maintain homeostasis and thrombin-responsiveness, especially in case of injury to cardiomyocytes [1]. Previous studies have documented that the recovery-responsiveness to thrombin in a cell has two phases: the first begins within several minutes lasting for several hours. In this period, PAR1 is recycled continuously between the cell surface and endosome, and hence PAR1 trafficking in the cell is essential for a continuous and prompt cellular response [2]. The second phase of recovery occurs for up to 20 h. This phase required the re-synthesis and membrane targeting of the receptors [3]. Rab11, a small G-protein that belongs to the subfamily of Rab proteins (members of the Ras superfamily of small GTPases), is involved in regulating vesicle recycling of the classical G-protein-coupled receptors, and reportedly plays a key role in regulating the intracellular trafficking of PAR1 through distinct endosomal sorting mechanisms[4, 5].

Thrombin is a crucial molecule involved in acute myocardial infarction (AMI) pathogenesis and is responsible for thrombus formation. Thrombin generation during AMI is a continuing process, which extends beyond the acute phase of MI [6]. We have previously reported that post-AMI, PAR1 expression increases in the ischemic myocardial tissue, and thrombin-mediated PAR1 activation improves cardiac function [7, 8]. In addition, hypoxia is a major challenge for cardiomyocytes during AMI and reportedly inhibits PAR1 expression;hypoxia-induced PAR1 inhibition could be reversed by TRAP induction in endothelial cells [9, 10]. Therefore, we hypothesized that alteration in cardiac function in AMI patients is associated with TRAP-induced alteration in expressions of PAR1 and Rab11.

## Methods and materials

TRAP and TFA (trifluoroacetic acid) were purchased from Sigma Chemical Co. (Shanghai, China). The antibodies for Rab11A, Rab11B, and GAPDH were obtained from Santa Cruz Biotechnology (shanhai, China), while rabbit primary antibody to PAR1 was procured from ABclonal (Cat No: A5641) and Santa Cruz Biotechnology (Cat No: SC13503). BacMam 2.0 gene delivery system for early endosome (Cat No: C10586, Rab5 labeled with GFP, green light) was purchased from Thermo Fisher Scientific (Shanghai, China). The TX chemiluminescent detection kit was obtained from Amersham, USA. The siRNAs targeting Rab11A and Rab11B were procured from Genetimes Tech Inc, Shanghai, China, andlipofectamine RNAiMAX was obtained from Invitrogen, USA.

TRAP was dissolved in 0.1% TFA to form a 50 mM stock solution and further diluted to a final concentration of 0.005% for the experiments. TFA is the vehicle. NETN buffer composition includes 100 mM NaCl, 20 mM Tris-Cl (pH 8.0), 0.5 mM EDTA, and 0.5% (v/v) Nonidet P-40 (NP-40).

### Animal model

All experimental protocols complied with the Guide for the Care and Use of Laboratory Animals published by the US National Institutes of Health (NIH) and the Animal Care and Use Committees of Universities and hospitals. Spague–Dawley rats (male, 200–250 g) were used for all experiments. The rat AMI model was created as described previously [7, 8]. Briefly, each ratwas anesthetized with an intraperitoneal injection (I.P. injection) of ketamine/xylazine (75/5 mg/kg), intubated, and ventilated on room air throughout the procedure. Under sterile conditions, a leftthoracotomy was performed at the third intercostal space. The pericardium was opened, exposing the left atrial appendage and pulmonary cone in the heart. Gentle pressure wasapplied on the right side of the thorax to provide quick access to the heart. A suture was placed under the left coronary artery (LCA) between the pulmonary artery outflow tractand the left atrium. If the ST segment in lead I was elevated upon tightening of the suture but returned to normal when the suture was relaxed, then the suture was tightened and tiedusing a 6-0 sterile silk 5 min after i.v. administration. The animal model of AMI was considered successful if: (1) the color of the infarcted area changed from red to white, (2) the left atrium was enlarged just after the coronary artery was ligated, and (3) the ST segment was elevated by more than 0.2 mm after LCA ligation. Myocardial ischemia was confirmed bythe presence of regional cyanosis and ST segment elevation on the ECG. It was further confirmed by Evans blue perfusion after every experiment.

Sham-operated rats served as controls. All rats were euthanized after the experiments by anesthetization with 100% O_2_/5% isoflurane, followed by either decapitation or transcardial perfusion with 0.9% saline containing 4% formaldehyde, depending on the protocol.

### Measurement of cardiac function

For left ventricular (LV) cannulation, all animals were adequately anesthetized, intubated in a supine position, and ventilated on room air with a small animal ventilator. To find the commoncarotid artery (CCA), the right CCA sheath was separated continuously, and the vagus nerve was freed. An attempt was made to insert the tube into the LV via the CCA. The LV pressurewas monitored by the tube. If the LV diastolic BP rapidly fell to near zero, then the tube was considered to have entered the LV.

After the tube was fixed, it was connected to a tension transducer and the BL-420 biological function experimental system (Chengdu Taimeng Technology Co, Ltd. China). Hemodynamicparameters of the LV, including the LV systolic pressure (LVSP max), LV end-diastolic pressure (LVEDP), and the rise and fall rates in the LV pressure (dp/dt max and dp/dt min, respectively),were obtained. Cardiac functional parameters were consecutively recorded within 2 h, at1 minute before and after intervention, and 1, 5, 10, 20, 40 min after LCAligation. To prevent blood from clotting in the tube, before each data point was recorded, the tube was washed with 200 µl of low-concentration heparin solution (12,500 U heparin in 500 ml of saline) for 5 s. To determine the cardiac functional parameters, each segment constituted 20 waves, with a 10-s interval between segments. For each time point, the average of three segments was used to determine the average cardiac functional parameter.

### Cardiomyocyte culture and hypoxia induction

All experimental protocols were performed in strict accordance with the ‘Guide for the Care and Use of Laboratory Animals’ published by the United States National Institutes of Health and the Institutional Animal Care and Use Committee of Hospitals and Universities. The primary cardiomyocyte culture was established as described in a previous report [11]. Neonatal Sprague–Dawley male rats (1–2 days old) were decapitated using sterile scissors (straight), and the chest opened along the sternum to allow access to the chest cavity and the heart. The hearts were isolated under sterile conditions and washed thrice with phosphate buffer saline (PBS). The ventricles were excised, minced into small pieces, and incubated for 1 h in a solution containing 100 mM NaCl, 10 mM KCl, 1.2 mM KH_2_PO_4_, 4.0 mM MgSO_4_, 50 mM taurine, 20 mM glucose, 10 mM HEPES, 2-mg/ml collagenase type II, 2-mg/ml pancreatin, and 1% penicillin–streptomycin. Subsequently, the detached cells were collected in 15-ml Falcon tubes and centrifuged at 1000 g for 5 min. The pellet was resuspended in DMEM containing 10% fetal bovine serum and 1% penicillin–streptomycin, and cultured in a flask at 37 °C in a humidified atmosphere of 5% CO_2_ and 95% air for 1 h to remove fibroblasts. The cardiomyocytes were then plated on 35-mm cell-culture dishes at a density of 2 million cells and cultured for 72 h. For the hypoxic environment, the cardiomyocytes were placed in a modular incubator chamber (MIC-101; Billups-Rothenberg Inc., CA, USA), flushed with a mixture of 1% O_2_, 5% CO_2_, and 94% N_2_, and incubated at 37 °C. Cells were then harvested at different time points as indicated [12].

### siRNA transfection

siRNA was obtained from Genetimes Tech Inc. (Shanghai, China) and the sequences used are described in Supplement1. A negative control siRNA was designed with the same GC ratio and without any known target in the rat genome. To confirm knockdown of Rab11a and Rab11b, siRNA sequences against Rab11a, Rab11b siRNA that exhibited at least 90% knockdown wereselected for the subsequent experiments.

Cultured cardiomyocytes were seeded in plates at a density of 6 × 10^5^ cells/well. After 24 h, the cells were incubated with a mixture of 6 pmol siRNAs above and 1 μl lipofectamine RNAiMAX (Invitrogen, CA) in 100 μl serum-free DMEM at 37 °C for 36 h. The transfection efficiency was examined by western blot analysis.

### Western blotting

The detailed procedure for western blotting has been described in a previous report [7, 8]. Briefly, cardiomyocytes were washed with ice-cold PBS and lysed in 1% SDS lysis buffer. These samples were then centrifuged at 4 °C and 15,000× *g* for 20 min. Their supernatants were mixed with a 5× loading buffer comprising of 0.25 M Tris–HCl (pH 6.8), 15% SDS, 50% glycerol, 25% β-mercaptoethanol, and 0.01% bromophenol blue, and then heated at 100 °C for 5 min. Proteins (30 µg/lane) were separated on a 15% gradient SDS–polyacrylamide gels and then transferred to polyvinylidene difluoride membranes using Mini Trans-Blot Cell (Bio-Rad, Hercules, CA). Membranes were cut based on molecular weights of target proteins and were blocked using a 5% bovine serum albumin at 4 °C for 1 h and then incubated with primary antibodies against Rab11a (1:1000), Rab11b (1:1000), PAR1 (1:750), and GAPDH (1:5000) (all antibodies purchased from Santa Cruz Biotechnology, TX) at 4 °C overnight in TBS with 0.1% Tween 20 (TBS-T) and 5% nonfat dry milk. After washing thrice with TBS-T, membranes were incubated for 1 h with horseradish peroxidase-conjugated secondary antibody (1:10,000 dilution; Pierce Biotechnology) and then washed again with TBS-T. Membranes were developed using enhanced chemiluminescence (Amersham, Arlington Heights, IL), and the results were analyzed using the DOG Gel2000 imaging system (Bio-Rad).

### Immunostain and confocal microscopy

Primary cultured cardiomyocytes were plated in confocal culture dishes and then transfected with siRNA against Rab11a, Rab11b, and their negative controls. Early endosomes were detected with BacMam 2.0 gene delivery system (Cat No: C10586, Rab5 labeled with GFP, green light; Thermo Fisher Scientific, Shanghai, China), and using the manufacturer’s instructions. The medium of cells in the confocal culture dishes was supplemented with cell-light reagent and incubated overnight (> 16 h) before fixing with 4% paraformaldehyde for 10 min. Thereafter, these cells in the confocal culture dishes were permeabilized with 0.1% TritonTM X-100 for 10 min, blocked with 1% BSA for an hour, and then labeled with Rabbit primary antibody to PAR1 (purchase from ABclonal, 1:1000) overnight at 4 °C. Goat anti-rabbit IgG(H + L) secondary antibody, Texas Red was used at a concentration of 4 µg/ml in PBS containing 0.2% BSA for 45 min at room temperature. The nuclei were stained with DAPI (Green).

### Statistical analysis

Data are reported as means ± standard errors of the mean (SEMs). Differences between means at different time points before and after LCA ligation (a repeated measures analysis) were evaluated by using a linear mixed effects model with a random effect for each anima performed using SAS Statistical Software (version 9.2, SAS Institute Inc.). Other in vitro data were analyzed using SPSS v25.0. while the experimental and control groups were compared using one-way ANOVA, followed by Bonferroni post-hoc testing in case of significance. Differences with *p* < 0.05 were considered statistically significant.

## Results

### Effect of TRAP induction on cardiac function of normal rats and rats with AMI

**Fig. 1:**
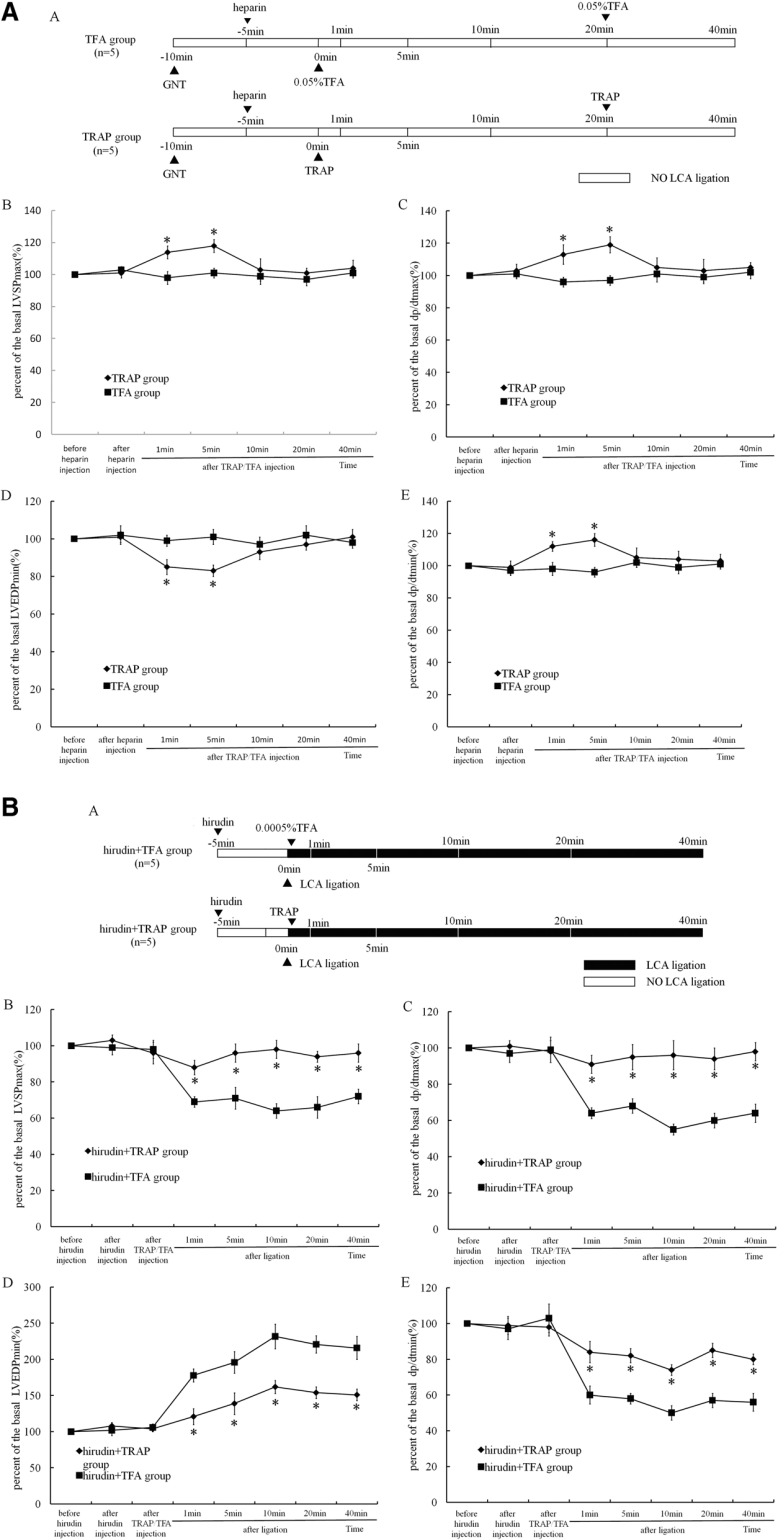
Effect of TRAP on cardiac function of normal rats and rats with acute myocardial infarction (AMI). **A**: **A**: Experimental protocol: Sprague–Dawley rats were divided into two groups with five normal animals per group: TRAP group and vehicle group (TFA). GNT (10 mg/kg) was injected by I.P. for 10 min and heparin (70 U/kg) was injected into the right iliac vein 5 min before the first injection of TRAP (20 mM,400 µl) and its vehicle TFA (0.05%, 400 µl). TRAP (20 mM, 400 µl) and TFA (0.05%, 400 µl) were injected again 20 min after the first injection. **B** and** C**: Percent changes in LVSP_max_ and dp/dt_max_ from basal values at different time points in each group. **D** and **E**: Percent changes in LVEDP and dp/dt_min_ from basal values at different time points in each group. Data is shown in mean ± SEM. *P < 0.05 for TFA versus TRAP group. **B**: **A**: Experimental protocol. Sprague–Dawley rats were divided into two groups of five animals each: Hirudin + TFA and hirudin + TRAP. Hirudin was administered intravenously via the left iliac vein 5 min before LCA ligation, followed by either TFA (0.0005%, 50 µl) or TRAP (100 µM, 50 µl) local injection in the infarcted area of left ventricles just after LCA ligation. **B** and **C**: Percent changes in LVSP_max_ and dp/dt_max_ from basal values at different time points in each group. **D** and** E**: Percent changes in LVEDP and dp/dt_min_ from basal values at different time points in each group. Data is shown in the mean ± SEM. *P < 0.05 for hirudin + TFA versus hirudin + TRAP group

As shown in Fig. 1A, for normal rats, compared to the vehicle (TFA)-injected rats, the first injection of TRAP (20 mM, 400 µl) increased LVSP _max,_ dp/dt_max,_ and dp/dt_min_, and decreased LVEDP_min_ to their peaks at 5 min after induction. All these parameters reverted back to normal within 10 min. The second injection of TRAP did not have any effect on cardiac function.

On the other hand, in rats with AMI,the right iliac vein was injected with 200 U/kg hirudin (to eliminate thrombin) or vehicle(0.01% mannitol) 5 min before LCA ligation (Fig. 1B). Compared to its vehicle, local injection of TRAP ameliorated AMI, decreased LVSP _max_, dp/dt_max,_ and dp/dt_min_, and increased LVEDP_min_ within 40 min.

### Effect of TRAP on PAR1 protein expression in primary cultured cardiomyocytes under normoxic conditions

**Fig. 2:**
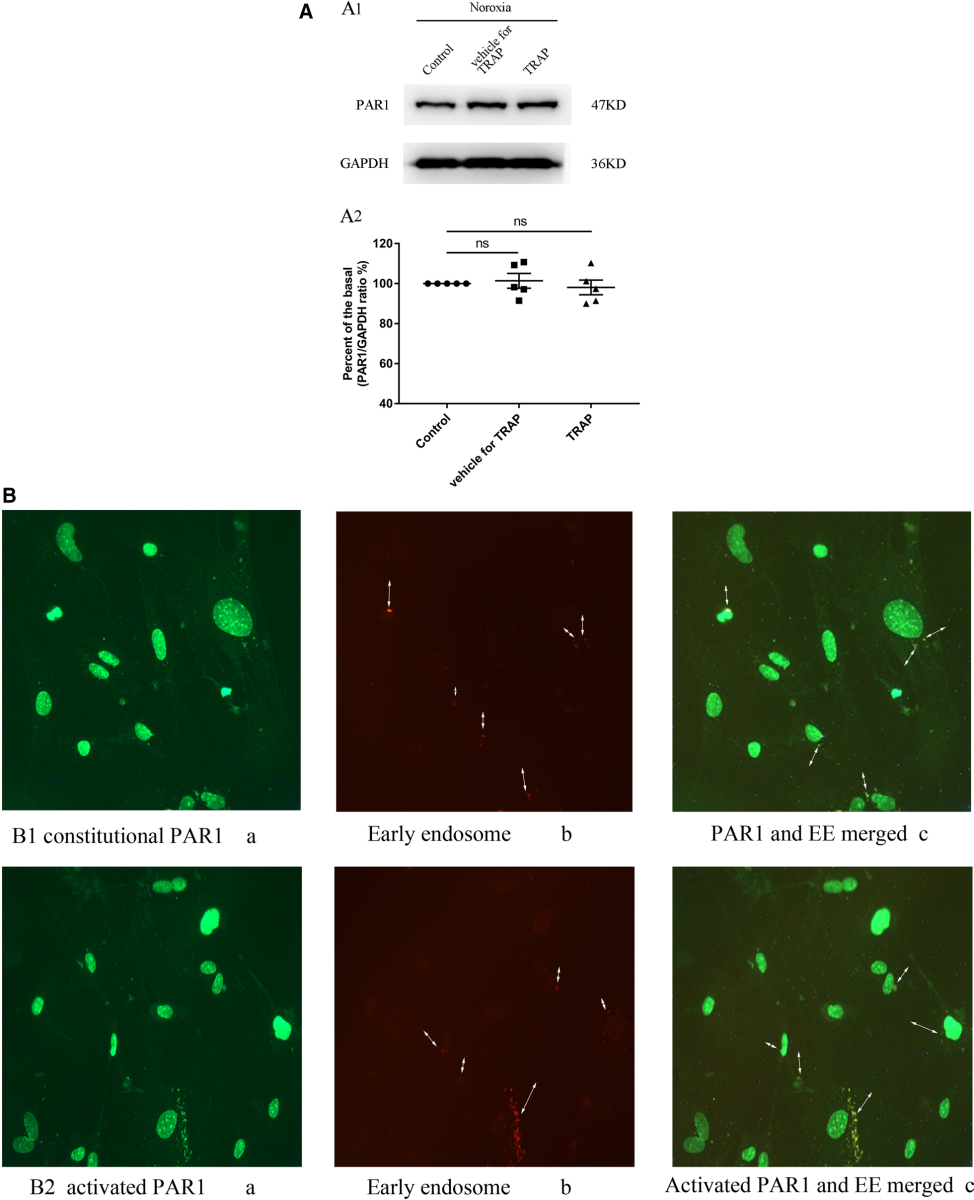
Effect of TRAP on primary cultured cardiomyocytes under the normoxic condition. **A**: **A1**: Representative western blot of PAR1 expression in primary cultured cardiomyocytes treated with TRAP (100 µmol/L) for 3 h; **A2**: Percent of basal of the ratio of PAR1 intensities to GAPDH intensity in western blot (n = 5). *P < 0.05 versus the control (PBS intervention). **B**: TRAP-induced colocalization of PAR1 with early endosome in primary cultured cardiomyocytes under the normoxic condition. Confocal microscopy showed that the PAR1-protein positive signals were green in color and the early endosome positive signals were red. **B1**: **a**: The green-colored positive signal of PAR1 located in the surface, cytoplasm, and nucleus of cardiomyocytes; **b**: The red-colored positive signal of early endosome located in the cytoplasm of cardiomyocytes; **c**: Co-localization (yellow color) of PAR1 and early endosome in the merged image. **B2**: **a**: The green-colored positive signal of PAR1 located in the surface, cytoplasm, and nucleus of cardiomyocytes treated with TRAP (100 µmol/L) for 3 h; **b**: The red-colored positive signal of early endosome located in the cytoplasm of cardiomyocytes treated with TRAP (100 µmol/L) for 3 h; **c**: Co-localization (yellow color) of PAR1 and early endosome in the merged image

To test the effect of PAR1 activation on PAR1 protein expression in cardiomyocytes, primary cultured cardiomyocytes were cultured under the normoxic condition and treated with TRAP (100 μmol/L). As illustrated in Fig. 2A, PAR1 protein expression was not affected by TRAP induction. This result demonstrates no effect of TRAP induction on PAR1 expression under normoxia. However, immunostaining for PAR1 and early endosomes in cardiomyocytes under normoxic conditions indicates a TRAP-induced redistribution of PAR1 (Fig. 2B). Under normoxic conditions, PAR1 was partly co-localized with early endosome in the cytoplasm, the surface, and the nucleus of cardiomyocytes. This finding indicated that TRAP seemingly stimulated PAR1 expression in the early endosome phase of cytoplasm, along with a reduced PAR1 level on the surface of cardiomyocytes.

### Effect of hypoxia on PAR1 expression in primary cultured cardiomyocytes

**Fig. 3:**
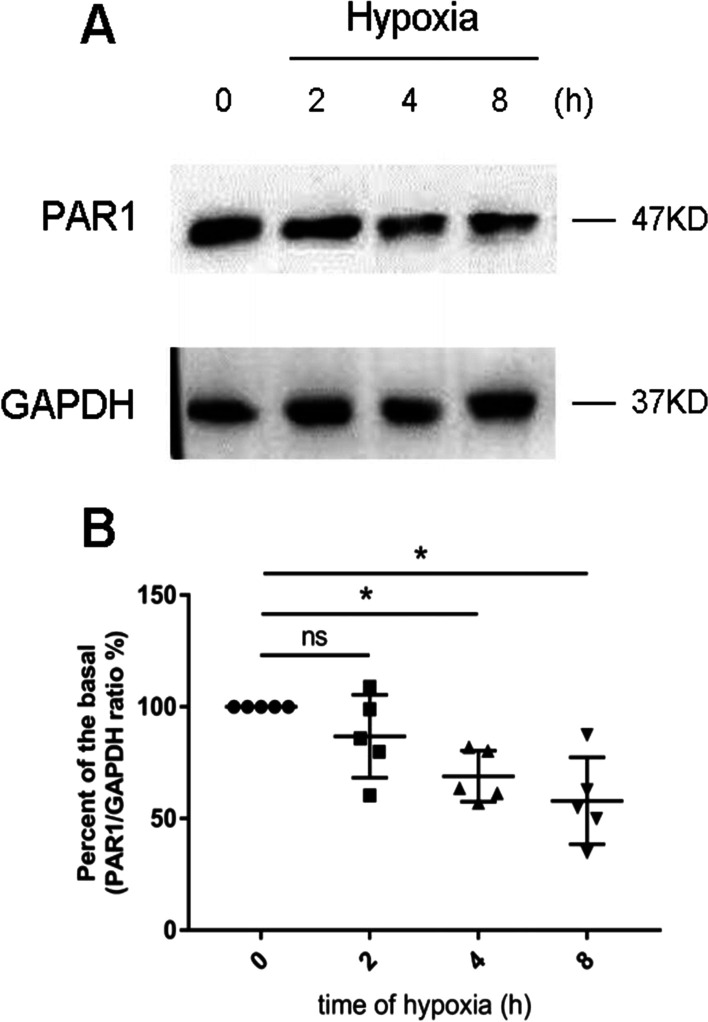
Primary cardiomyocytes were cultured under hypoxic conditions for the indicated durations before harvesting. **A** Representative western blot analysis of PAR1 expression in primary cultured cardiomyocytes exposed to hypoxia at indicated time points. **B** Percent of basal of the ratio of PAR1 intensities to GAPDH intensity in western blot (n = 5). *P < 0.05 versus the normoxic group (0 point)

Primary cardiomyocytes were cultured in a MIC-101 culture under hypoxic conditions for indicated durations before harvesting. PAR1 protein expression gradually decreased during hypoxia (Fig. 3), compared with the normoxic/basal group. These results necessitate deliberation on what could be the mechanism behind the hypoxia-induced decrease in PAR1 expression.

### Effect of hypoxia on Rab11A and Rab11B expressions in the primary cultured cardiomyocytes

**Fig. 4:**
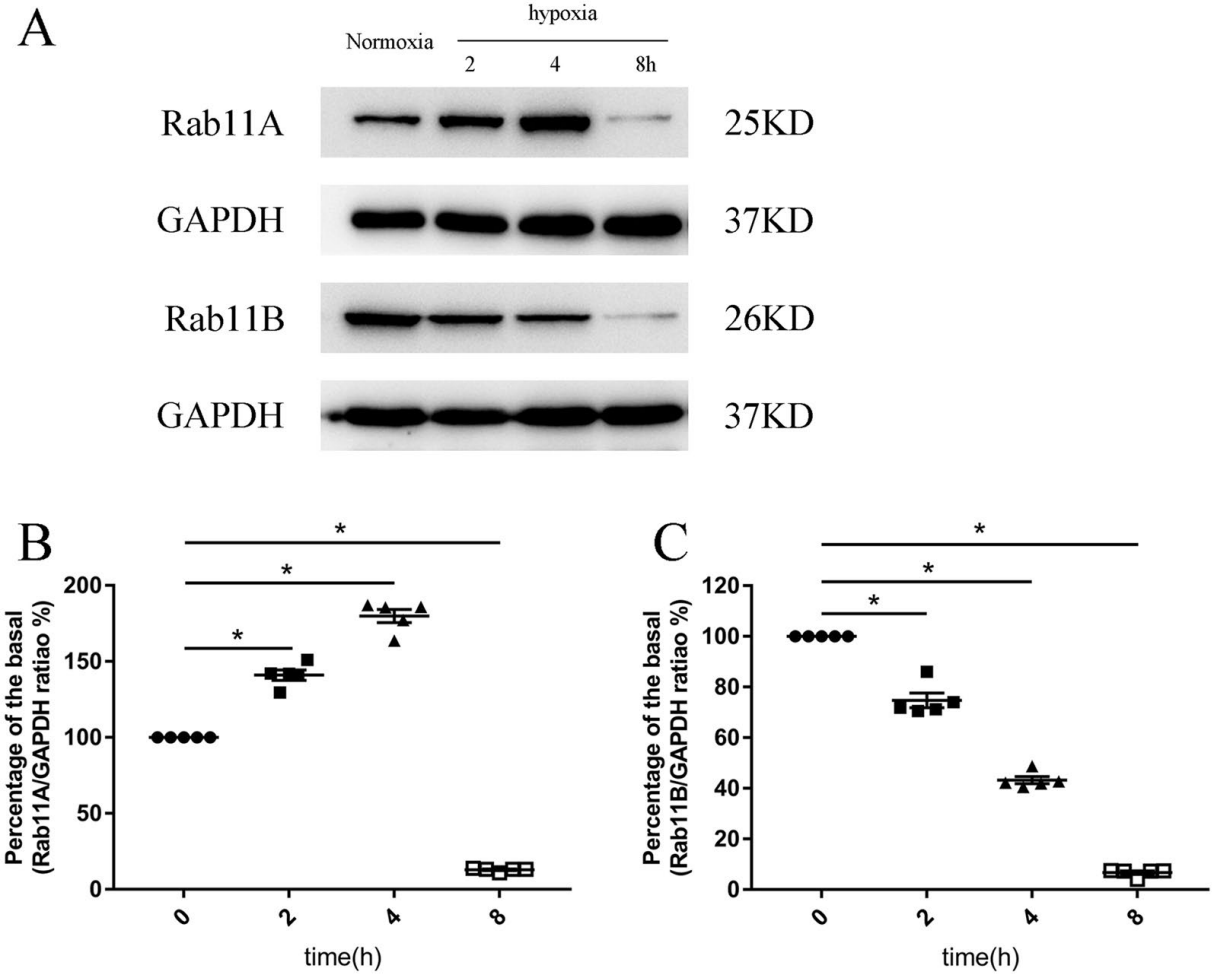
The effect of hypoxia on expressions of Rab11A and Rab11B in primary cultured cardiomyocytes. **A** Representative western blots of Rab11 A and Rab11B expressions in primary cultured cardiomyocytes exposed to hypoxia at different time points; **B** and** C**: Percent of the basal of the ratio of Rab11 A (**B**) and Rab11B (**C**) intensity to GAPDH intensity in western blot (n = 5). *P < 0.05 versus the normoxic group (0 point)

PAR1 protein levels in cells are maintained by the balance between PAR1 degradation and replenishment, and constitutively-cycled PAR1 is regulated by Rab11 in non-cardiac cells [4]. Therefore, we attempted to understand the effect of hypoxia on Rab11A and Rab11B expressions in primary cultured cardiomyocytes. As shown in Fig. 4, Rab11A levels increased and reached a peak during the first 4 h of hypoxia, and then, decreased. Contrarily, Rab11B levels continuously decreased during the hypoxia. Since Rab11A and Rab11B are responsible for the degradation and recycling of PAR1 in cells, respectively [4], our result justifies the hypoxia-induced decrease in PAR1 expression in cardiomyocytes.

### Effect of TRAP induction on PAR1 protein expression in primary cultured cardiomyocytes during hypoxia

**Fig. 5:**
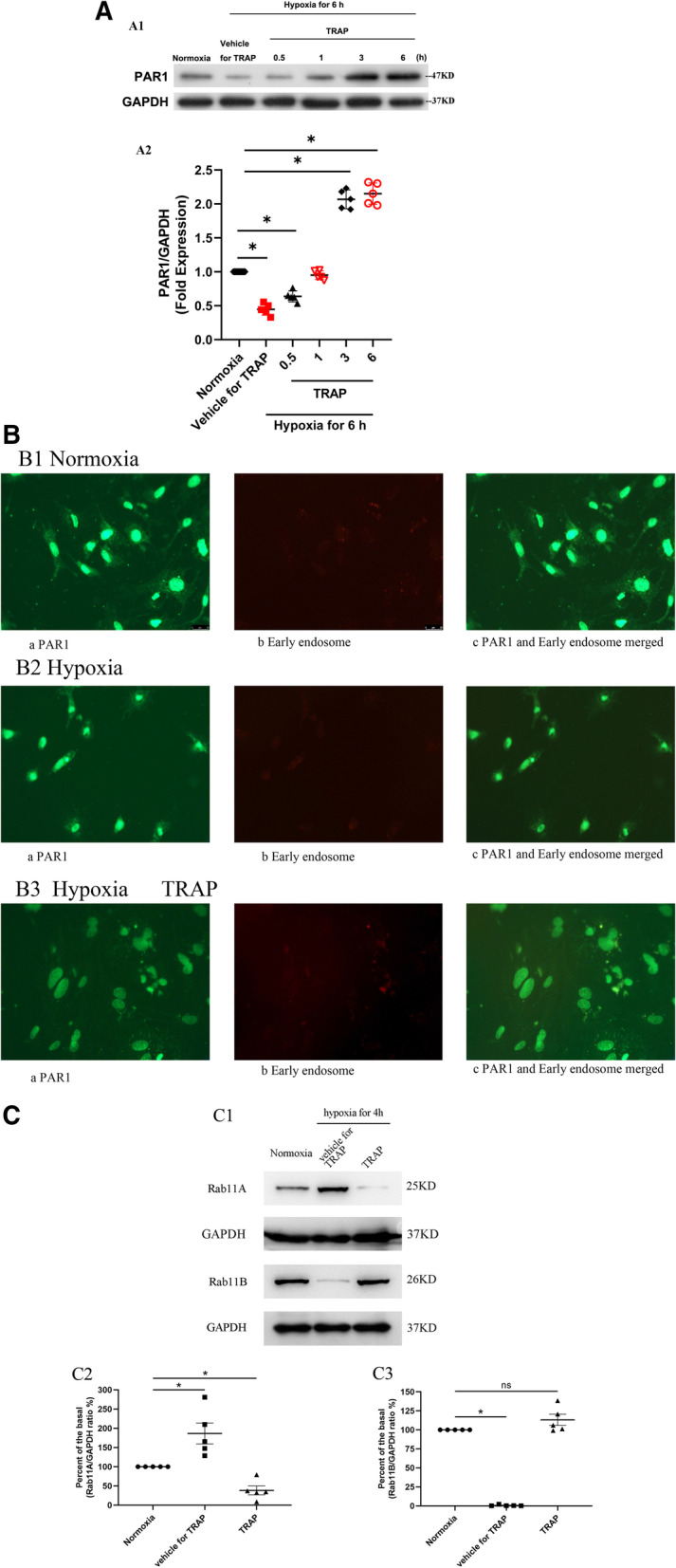
**A**: Effect of TRAP on PAR1 expression in primary cultured cardiomyocytes after a 6-h exposure to hypoxia. **A1**: Representative western blots of PAR1 expression in primary cultured cardiomyocytes treated with TRAP (100 µmol/L) for another 0 h, 0.5 h, 1 h, 3 h, and 6 h, after 6-h, 5.5-h, 5-h,and 3-h exposure to hypoxia. **A2**: Percent of basal of the ratio of PAR1 intensities to GAPDH intensity in western blot (n = 5); *P < 0.05 versus the normoxic group (zero point). **B**: **B1**: **a**: The green-colored positive signal of PAR1 located on the surface, cytoplasm, and nucleus of cardiomyocytes under normoxic condition; **b**: The red-colored positive signal of early endosome located in the cytoplasm of cardiomyocytes under normoxic condition; **c**: Co-localization (yellow color) of PAR1 and early endosome in the merged image. **B2**: **a**: The green-colored positive signal of PAR1 located on the surface, cytoplasm, and nucleus of cardiomyocytes under hypoxia; **b**: The red-colored positive signal of early endosome located in the cytoplasm of cardiomyocytes under hypoxia; **c**: Co-localization (yellow color) of PAR1 and early endosome in the merged image. **B3**: **a**: The green-colored positive signal of PAR1 located on the surface, cytoplasm, and nucleus of cardiomyocytes treated with TRAP (100 µmol/L) for 3 h under the hypoxia; **b**: The red-colored positive signal of early endosome located in the cytoplasm of cardiomyocytes treated with TRAP (100 µmol/L) for 3 h under hypoxia; **c**: Co-localization (yellow color) of PAR1and early endosome in the merged image. **C**: Effect of TRAP on PAR1 expression in primary cultured cardiomyocytes exposed to hypoxia. **C1**: Representative western blots of Rab11A and Rab11B expression in primary cultured cardiomyocytes under hypoxia for 3 h, followed by the addition of TRAP (100 μmol/L) or vehicle for TRAP and incubation for another 1 h under hypoxia; **C2**: Percent of the basal of the ratio of Rab11 A intensity to GAPDH intensity in western blot (n = 5). **C3**: Percent of the basal of the ratio of Rab11 B intensity to GAPDH intensity in western blot (n = 5). *P < 0.05 versus the normoxic group

To test the effect of PAR1 activation on PAR1 protein expression in cardiomyocyte during hypoxia, primary cultured cardiomyocytes were cultured under hypoxia for 6 h, 5.5, 5, and 3 h, followed by the addition of TRAP (100 μmol/L) and incubation for another 0, 0.5, 1, 3, and 6 h as indicated before harvesting. As shown in Fig. 5A, the PAR1 expression in both vehicle and TRAP-treated rats differed significantly under hypoxic conditions compared to that under normoxic conditions (*p* < 0.05). PAR1 protein expression at 6 h of hypoxia in the vehicle for the TRAP group as against the normoxic controls was at 44.7% ± 3.9% (*p* < 0.05 vs. normoxic group). For the TRAP group versus normoxic group at TRAP-treated time-points, the PAR1 expression at 0.5, 1, 3, and 6 h was at 63.9% ± 3.7%*, 95.1% ± 4.1%, 206.8% ± 8.2%*, and 215.2% ± 7.2%* (n = 5, *p < 0.05 vs. normoxic group) of that in the normoxic group. Additionally, immunostaining results under hypoxia indicated that hypoxia decreased the PAR1 expression both on the surface and in early endosomes of cells. Furthermore, TRAP induction led to a reduced existence of PAR1 on the surface but an increased expression in the early endosome of cells (Fig. 5B). Thus, hypoxia is important for the TRAP-induced increase in PAR1 expression in cardiomyocytes.

We had hypothesized that PAR1 activation affects the Rab11A and B expression in cardiomyocytes under hypoxia, for which the effect of TRAP on Rab11 protein expression under hypoxia was also tested. Primary cultured cardiomyocytes were subjected to hypoxia for 3 h, followed by the addition of TRAP (100 μmol/L) and the vehicle for TRAP, and incubated for another hour before harvesting. TRAP-induction decreased hypoxia-induced Rab11A expression after 4 h of hypoxia (Fig. 5C). On the contrary, TRAP induction led to an increase in hypoxia-decreased Rab11B expression after 4 h of hypoxia. Since Rab11A and Rab11B levels in cardiomyocytes were barely detectable at 8 h after hypoxia (Fig. 4), our result could partially explain the TRAP-induced PAR1 expression through modification in Rab11A and Rab11B expressions in cardiomyocytes under hypoxia.

### Role of Rab11 in TRAP-induced PAR1 expression in primary cultured cardiomyocytes under hypoxia

**Fig. 6:**
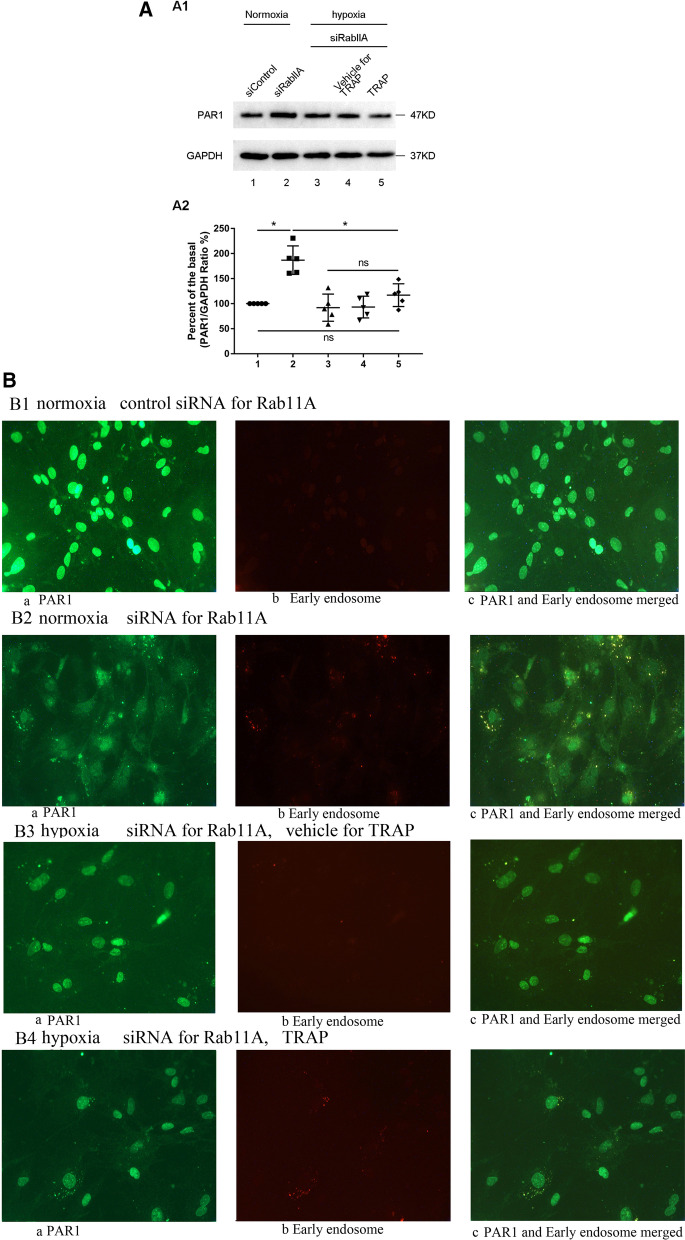

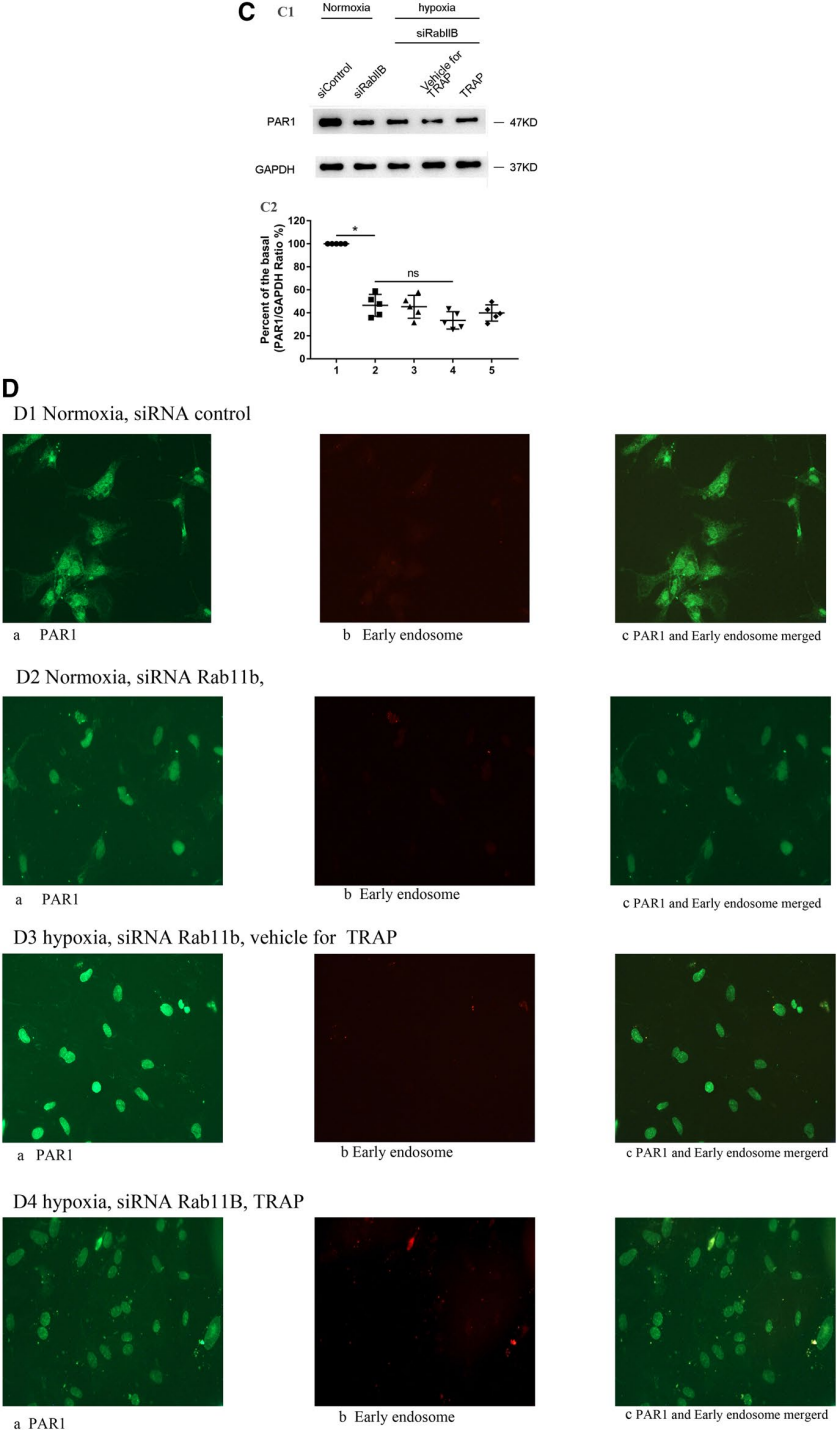
The role of Rab11A and Rab11B in TRAP-induced PAR1 expression in cardiomyocytes during hypoxia. **A**: **A1**: Representative western blot of TRAP-induced PAR1 expression in primary cultured cardiomyocytes transfected by siRNA to Rab11A and its control siRNA under hypoxia for 5 h followed by the addition of TRAP or its vehicle for another 1 h; **A2**: Percent of the basal of the ratio of PAR1 intensity to GAPDH intensity in western blot (n = 5). *P < 0.05 versus the normoxic group. **B**: Confocal microscopy showed that PAR1 protein positive signals were green in color and early endosome positive signals were red in color. **B1**: Co-localization of PAR1 expression with early endosome in primary cultured cardiomyocytes transfected by si Rab11A under normoxia;** a**: The green-colored positive signal of PAR1 located on the surface, cytoplasm, and nucleus of cardiomyocytes under the normoxic condition;** b**: The red-colored positive signal of early endosome located in the cytoplasm of cardiomyocytes under the normoxic condition; **c**: Colocalization (yellow color) of PAR1 and early endosome in the merged image. **B2**: TRAP-induced colocalization of PAR1 with early endosome in primary cultured cardiomyocytes transfected by si Rab11A under normoxia;** a**: The green-colored positive signal of PAR1 located in the surface, cytoplasm, and nucleus of cardiomyocytes under the normoxic condition; **b**: The red-colored positive signal of early endosome located in the cytoplasm of cardiomyocytes under the normoxic condition; **c**: Co-localization (yellow color) of PAR1 and early endosome in the merged image. **B3**: TRAP-induced PAR1 expression in primary cultured cardiomyocytes transfected by si Rab11A under hypoxia for 5 h followed by the addition of TRAP vehicle for another 1 h. **a**: The green-colored positive signal of PAR1 located on the surface, cytoplasm, and nucleus of cardiomyocytes treated with TRAP vehicle under the hypoxic condition; **b**: The red-colored positive signal of early endosome located on the cytoplasm of cardiomyocytes treated with TRAP vehicle under the hypoxic condition;** c**: Co-localization (yellow color) of PAR1and early endosome in the merged image. **B4**: TRAP-induced PAR1 expression in primary cultured cardiomyocytes transfected by si Rab11A under hypoxia for 5 h, followed by the addition of TRAP (100 µmol/L) for another 1 h. **a**: The green-colored positive signal of PAR1 located on the surface, cytoplasm, and nucleus of cardiomyocytes; **b**: The red-colored positive signal of early endosome located on the cytoplasm of cardiomyocytes; **c**: Co-localization (yellow color) of PAR1and early endosome in the merged image. **C**: **C1**: Representative western blot of TRAP-induced PAR1 expression in primary cultured cardiomyocytes transfected by si Rab11B and its control siRNA under hypoxia for 5 h followed by the addition of TRAP or its vehicle for another 1 h; Bottom: **C2**: Percent of the basal of the ratio of PAR1 intensity to GAPDH intensity in western blot (n = 5). *P < 0.05 versus the normoxic group. **D**: Confocal microscopy showed that PAR1 protein positive signals were green in color and early endosome positive signals were red in color. **D1**: TRAP-induced colocalization of PAR1 with early endosome in primary cultured cardiomyocytes transfected by si Rab11B under normoxia;** a**: The green-colored positive signal of PAR1 located on the surface, cytoplasm, and nucleus of cardiomyocytes under the normoxic condition; **b**: The red-colored positive signal of early endosome located in the cytoplasm of cardiomyocytes under the normoxic condition; **c**: Co-localization (yellow color) of PAR1 and early endosomes in the merged image. **D2**: TRAP-induced colocalization of PAR1 with early endosome in primary cultured cardiomyocytes transfected by si Rab11B under normoxia; **a**: The green-colored positive signal of PAR1 located on the surface, cytoplasm, and nucleus of cardiomyocytes under the normoxic condition;** b**: The red-colored positive signal of early endosome located in the cytoplasm of cardiomyocytes under the normoxic condition; **c**: Co-localization (yellow color) of PAR1 and early endosomes in the merged image. **D3**: TRAP-induced PAR1 expression in primary cultured cardiomyocytes transfected by si Rab11B under hypoxia for 5 h followed by the addition of TRAP vehicle for another 1 h.** a**: The green-colored positive signal of PAR1 located on the surface, cytoplasm, and nucleus of cardiomyocytes treated with TRAP vehicle under the hypoxic condition; **b**: The red-colored positive signal of early endosomes located in the cytoplasm of cardiomyocytes treated with TRAP vehicle under the hypoxic condition; **c:** Co-localization (yellow color) of PAR1and early endosome in the merged image. **D4**: TRAP-induced PAR1 expression in primary cultured cardiomyocytes transfected by si Rab11B under hypoxia for 5 h followed by the addition of TRAP (100 µmol/L) for another 1 h.** a**: The green-colored positive signal of PAR1 located on the surface, cytoplasm, and nucleus of cardiomyocytes; **b**: The red-colored positive signal of early endosome located in the cytoplasm of cardiomyocytes; **c**: Co-localization (yellow color) of PAR1and early endosome in the merged image

The effect of TRAP on Rab11 protein expression was tested in cardiomyocytes during hypoxia. Therefore, we hypothesized that the inhibition of Rab11A and Rab11B affected the PAR1 induced-PAR1 expression. The primary cultured cardiomyocytes transfected with siRNA to Rab11A were cultured under hypoxia for 3 h, followed by the addition of TRAP (100 μmol/L) and vehicle for TRAP for another 3 h before harvesting. As depicted in Fig. 6A, PAR1 expression in cardiomyocytes transfected with Rab11A siRNA increased under normoxic conditions. However, TRAP induction did not trigger PAR1 expression under hypoxia in cardiomyocytes transfected with Rab11A siRNA.

As shown in Fig. 6B, immunostaining of cardiomyocytes containing Rab11A siRNA showed that PAR1 levels increased both on the cell surface and in the early endosome in the cytoplasm under normoxic conditions (Fig. 6B2). However, no significant changes were observed in total PAR1 expression level post TRAP stimulation under hypoxic conditions. This finding might be attributed to the fact that its levels decreased on the cell surface despite an increase in PAR1 levels in the cytoplasm (Fig. 6B4). Thereafter, the primary cultured cardiomyocytes transfected with Rab11B siRNA were cultured under hypoxia for 3 h, followed by the addition of TRAP (100 μmol/L) and vehicle for TRAP for another 3 h before harvesting. Surprisingly, we observed a significant decrease in PAR1 expression levels in cardiomyocytes transfected with Rab11B siRNA under both normoxia and hypoxia (Fig. 6C). Furthermore, TRAP stimulation did not decrease PAR1 expression in hypoxic cardiomyocytes transfected with Rab11B siRNA.

Similarly, immunostaining with PAR1 and early endosome in cardiomyocytes knocked out Rab11B with its siRNA, indicating that PAR1 decreased both on the surface of cells and in the early endosome of the cytoplasm under both normoxic and hypoxic conditions (Figs. 2, 6D1). TRAP induced PAR1 expression did not occur, but reduced PAR1 on the surface, and an increase in the cell cytoplasm could still be seen (Figs. 4, 6D3).

## Discussion

Our work showed that PAR1 activation with TRAP temporarily enhanced the cardiac function of normal rats for 10 min, after which it decreased. The second TRAP injection did not yield any significant changes.PAR1 activation proved the direct inotropic effect on heart; however, the effect of PAR1 activation on cardiac function is still controversial although the effective durations (within 10 min) were the same [13, 14]. PAR1 activation causes vessel constriction. Previously, Damiano et al. [14] showed that TRAP decreased the perfusion of the heart, and therefore, temporarily decreased the cardiac function within 10 min, following by reversion to normal cardiac function. To prevent vessel constriction in our experiments, GNT (10 mg/kg) was injected by I.P.to maintain the stable concentration of GNT in heart tissue treated with TRAP to retain vessel relaxation for 4 h [15]. TRAP-activated PAR1, which is different from thrombin-activated PAR1, is reversible and susceptible to degradation by lysosomes. Similarly, the unactivated receptor can be largely recycled back to the cell surface to maintain the effect of TRAP [1]. The temporary effect of TRAP on cardiac function might be attributed to the peak concentration of TRAP post-infusion or the receptor desensitization. The absence of any such effects of second TRAP injection on cardiac function further supported the inference of receptor desensitization. The desensitization of PAR1 involves the process of receptor internalization and transfer from endosomes to the lysosome.

The present study on cardiomyocytes revealed that PAR1 normally exists on the surface, cytoplasm, and cell nucleus. The total PAR1 protein levels in cardiomyocytes are very stable, and a balance of recycling, synthesis and degradation of PAR1 is achieved under normoxia, suggesting that these pathways coexist to maintain PAR1 recovery [2]. The present study shows that the TRAP stimulation had no effect on total PAR1 expression level in cardiomyocytes under normoxic conditions, but confocal microscopy indicated that PAR1 expression decreased on the surface, increased in the early endosome, and remained unchanged in the cell nucleus and cytoplasm. Since the only receptor on the cell surface can produce the biological effect, this redistribution explained the transient effect of PAR1 activation and no effect of the second dose of TRAP on cardiac function in normal rats. The activated PAR1 on the cell surface is possibly internalized and transferred to the early endosome, which sorts the receptor either back to the cell surface or endosome for storage or lysosome for degradation.

However, TRAP-induced PAR1 activation persistently reversed cardiac function in rats with AMI within 40 min. The ischemic area at risk and the area of necrosis of the rat AMI model reached stability at 40 min, which is the same at 24 h after coronary ligation [16]. This in vivo data indicated that PAR1 was continuously sensitive to TRAP. This finding indicates that TRAP-induced PAR1 activation was able to rescue PAR1 expression in cardiomyocytes under hypoxic conditions.

During hypoxia, total PAR1 expression decreased in the absence of the TRAP intervention. Higher levels of consumed PAR1 during hypoxia compared to replenished and constitutively cycled PAR1 resulted in decreased PAR1 expression and vice versa. The endocytic trafficking of PAR1 from the early endosome to the plasma membrane or lysosome is essential for recycling PAR1 [3]. Several Rab GTPases synergistically regulate the ER-Golgi transport, endocytosis, and trafficking of G-coupled receptors between early, late, and recycling endosomes and lysosomes [17]. For instance, Rab4 is involved in rapid recycling and Rab11 in slow recycling of vesicles from endosome to plasma membrane [18]. Rab11 is known to be a key regulator in the endocytic trafficking of many receptors, including PAR1. Members of the Rab11 family, including Rab11A, Rab11B, and Rab25 share high sequence similarities and are localized to the endosomal-recycling compartment (recycling endosome) [4, 19].

The recovery of PAR1 sensitivity in non-cardiac cells, such as fibroblasts, occurs over few minutes to several hours to ensure fast recovery of the PAR1 sensitivity, while the new receptor synthesis requires more than 20 h. Ellis et al. reported that it only took 1 h (less than 1 h was not tested) for thrombin and TRAP to increase PAR1 expression (at both mRNA and protein levels) in endothelial cells under hypoxia [9, 10]. However, Landau et al. reported a reversal of hypoxia-induced decrease in PAR1 mRNA expression within 48 h in hypoxic cardiomyocytes treated by TRAP [20]. Our current results showed that the TRAP-induced PAR1 activation could rescue PAR1 expression within 1 h in cardiomyocytes under hypoxia for 6 h. This finding could be attributed to the following reasons: (1) Different cells have different characteristics with different mechanisms. Sensitivity to oxygen and different growth factors in endothelial cells and cardiomyocytes is different from fibroblasts. Hence, the recovery time after PAR1 activation differs. (2) The experimental conditions were different. Landau et al. had treated cardiomyocytes with TRAP and thrombin for 48 h and then put them under 0.2% oxygen for 12 h. However, cardiomyocytes are very sensitive to oxygen, and hence, clinically, revascularization (recovery of normal oxygen) should be achieved as early as possible (the door to balloon time is limited to 90 min). With the passage of the first 12 h of AMI, the heart function is irreversibly damaged because of the extensive cell death, and cardiomyocytes are rarely known to regenerate. The experiments conducted in the present study reinforce the fact that not many cardiomyocytes can survive in 0.2% oxygen for 12 h. Hence, the resulting population of fibroblasts might outnumber the cardiomyocytes in the cultured rat ventricular cells in the aforementioned conditions. (3) The constitutive receptor recycle as well as the new receptor synthesis are presumably continuous and simultaneous processes occurring in physiological conditions. The PAR1 levels in cardiomyocytes are in a dynamic balance of degradation, synthesis, and recycling. PAR1 activation only enhances or decreases some steps of this process.

PAR1 is gradually degraded with increased Rab11A in cardiomyocytes during the hypoxia; however, the PAR1 expression in cardiomyocytes with siRab11A under a hypoxic environment did not change much compared with cardiomyocytes transfected with control siRNA. These results indicated that TRAP reversed the reduced PAR1 levels in hypoxia by inhibiting Rab11A expression, which is a key regulatory step for converting the early endosome into the late endosome [21]. Hence, intracellular accumulation of PAR1 results from a Rab11A-induced PAR1 breakdown by basal lysosomal degradation of the receptor. Our results revealed that PAR1 expression in cardiomyocytes with siRab11A increased under normoxia. This result confirms the role of Rab11A in the degradation of PAR1 in cardiomyocytes, in both normoxia and hypoxia. Rab11A also controls endosomal-lysosomal sorting and degradation of PAR1 in Hela cells [22]. However, we observed that hypoxia affects the PAR1 expression negatively in cardiomyocytes with knocked-out Rab11A and, similar to normoxic conditions, TRAP stimulation did not reverse this reduced expression but ensured higher PAR1 localization in the early endosome in these cardiomyocytes. This result indicated that PAR1 could be degraded via other minor pathways independent of Rab11A.

Furthermore, this study revealed that PAR1 is gradually degraded with a decrease in Rab11B expression in cardiomyocytes under hypoxia. Rab11B is localized to apical vesicles, distinct from a Rab11A compartment, and regulates vesicular trafficking through recycling of the endosomal compartment and the early endosomes to the trans-Golgi network and plasma membrane [23]. Rab11B is documented as a key regulator of recycling the constitutively internalized PAR1 back to the cell surface in Hela cells [4]. It also prevents the autophagic trafficking of PAR1 for degradation [24]. In the present study, PAR1 expression decreased in cardiomyocytes with siRab11B under both normal and oxygen-deficient conditions. TRAP stimulation was unable to reverse this decreased PAR1 expression under hypoxia combined with transfection of siRab11B, although it did result in a redistribution of PAR1 similar to the normal cardiomyocytes. Therefore, Rab11B is a critical factor for PAR1 in hypoxic cardiomyocytes not only for recycling it back to the cell surface but also for checking its degradation in cardiomyocytes.

*Clinical implication* Under normal physiological condition, thrombin concentration in blood and tissue is extremely low. When AMI or other thrombotic diseases occur, a large amount of thrombin is produced in the process of thrombosis and fibrinolysis. In the process of thrombosis, the massive produced thrombin changes fibrin monomers into fibrin polymer network which encapsulates the blood cells and thrombin itself, and then thrombus forms and thrombin loses its function. In the following process of fibrinolysis, thrombin is released from thrombus during the process of fibrin degradation [25]. Therefore, in AMI, thrombin is mainly produced in the local area of myocardial infarction. The effect of thrombin is also limited to the area of myocardial infarction. Once thrombin produced locally is infiltrated or in other places carried into normal myocardium, our results show that thrombin has limited effect on normal myocardium. It is important for the heart to maintain its normal function under pathological conditions. For example, thrombin can restore the myocardial contractility of the damaged area without affecting the normal myocardial contractility, which can ensure that the heart can coordinate contraction (synchronization) and restore cardiac function; otherwise, the un-synchronization heart would lose it function.

*Limitation* Our previous in vivo study showed that PAR1 expression in infarcted myocardial tissues peaked at 30 min and disappeared at 60 min after acute left coronary ligation of rat heart, which is different from our current in vitro data [8]. The stop blood supplies, including blood glucose, various nutritional factors and neurohumoral regulatory factors et al., of infarcted myocardial tissues are much more than hypoxia. Previous studies have reported that cultured cardiomyocytes could survive hypoxia for more than 40 min. The ischemic area at risk and the area of necrosis of rat AMI model reached stability at 40 min, which is the same as that at 24 h after coronary ligation [16]. Therefore cultured cardiomyocytes in hypoxia only mimic AMI, but not the whole process of AMI.

**Fig. 7:**
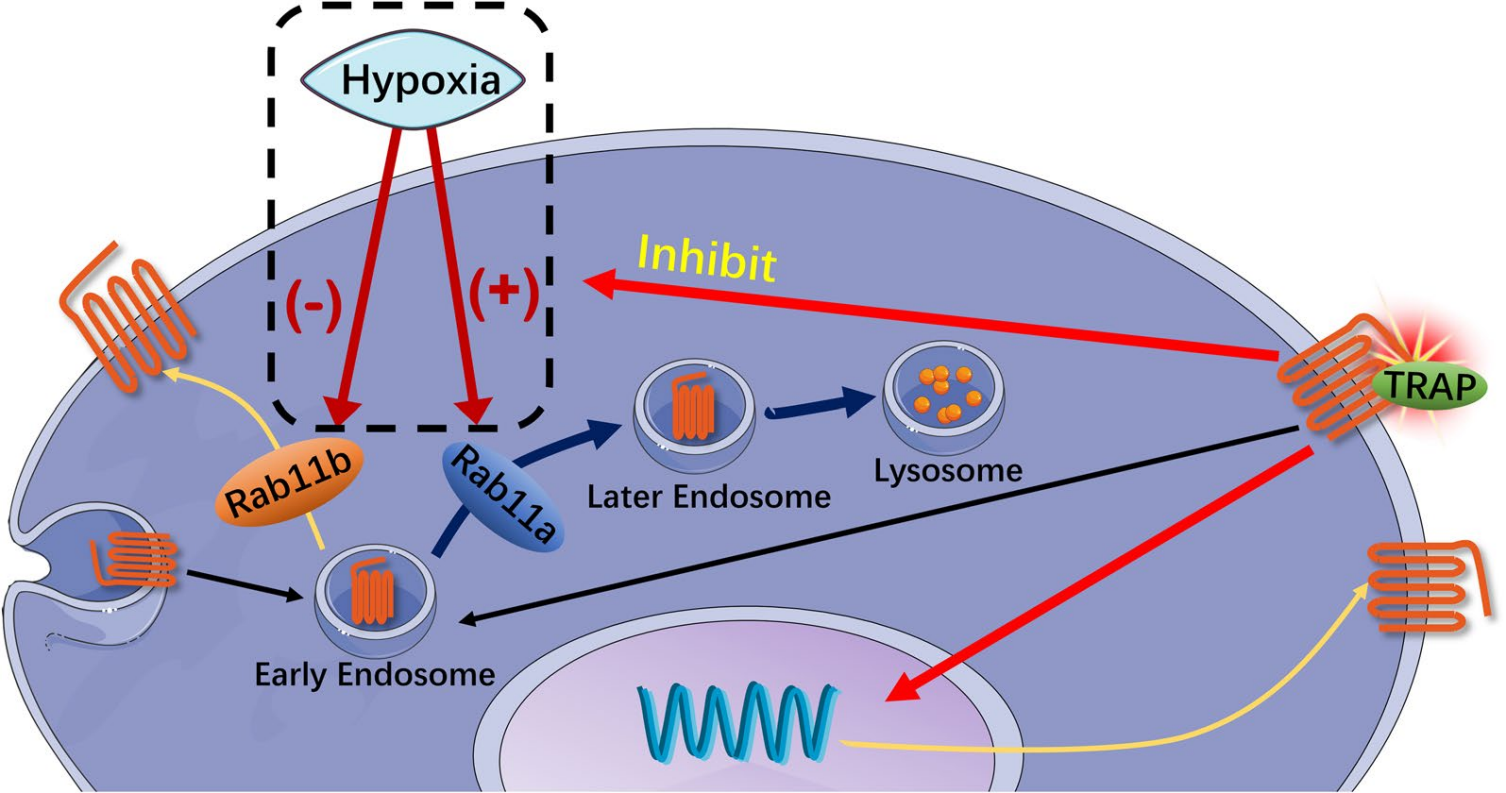
The related mechanisms of PAR1 activation during the AMI

To summarize, PAR1 activation only has a transient effect on cardiac function in normal rats but led to persistent improvement in rats with AMI. All related mechanisms are depicted in Fig. 7. TRAP activation of PAR1 in cardiomyocytes does not affect a change in the total PAR1 expression under normoxia, rather triggers a redistribution of PAR1 expression under both normal and oxygen-deficient conditions. TRAP stimulation successfully reverses the hypoxia-inhibited PAR1 expression in cardiomyocytes by inhibiting Rab11A and enhancing Rab11B expression.

## Supplementary Information

**Additional file 1.** Original gels for figures 2, 3, 4, 5, 6.

**Additional file 2.** Original gels for figures 4, 5, 6.

**Additional file 3.** The siRNA sequnces for Rab11A, Rab11B.

## Data Availability

The datasets used and/or analysed during the current study available from the corresponding author on reasonable request.

## Abbreviations

PAR1: Protease activated receptor 1
AMI: Acute myocardial infarction
TRAP: Thrombin receptor activated peptide
GNT: Glyceryl trinitrate
