# Supplementary material for: TRAP-induced PAR1 expression with its mechanism during AMI in a rat model
Source: BMC Cardiovasc Disord. 2023 Feb 21;23:97. doi: 10.1186/s12872-023-03118-w (PMC9942295; doi:10.1186/s12872-023-03118-w)
Supplement: Supplementary file 1 — Additional file 1. Original gels for figures 2, 3, 4, 5, 6. [file 12872_2023_3118_MOESM1_ESM.pdf]

we provided all the original gels of the paper. Since the research in different labs has spaned for more than 7 years and not every lab had modern equipments for all gels, the earlier membranes (transferred from gels) did not have any markers and for the reasons to save the antibodies, we usually didnot use the whole membranes (transferred from the gels) for the reaction with the antibodies, and we also did not keep the imagines of the whole membranes (transferred from the gels) (no journals in 3 years ago required the whole gel).

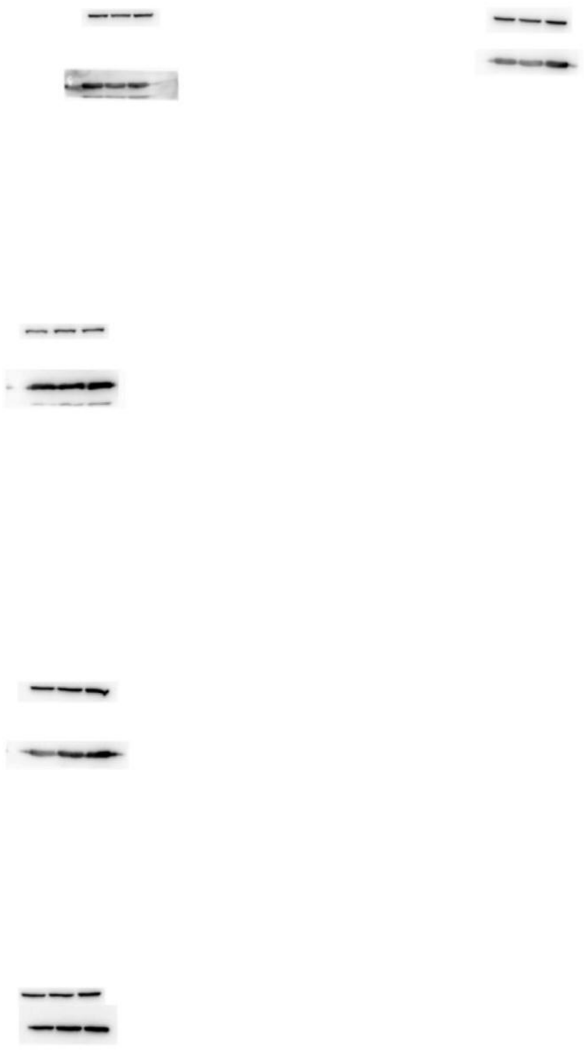

The original figures of Figure 2A(PAR1 37KD, GAPDH36KD)

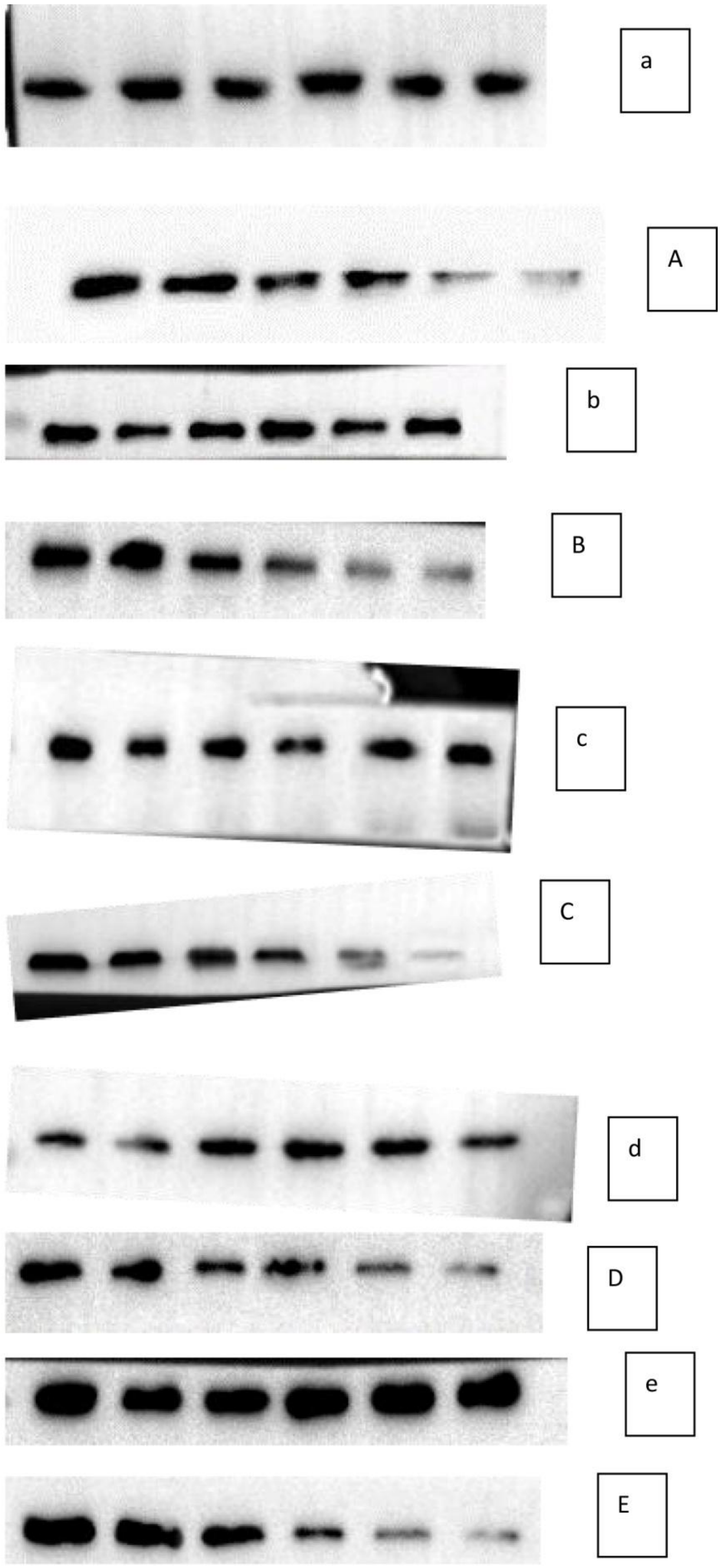

The original figures of Figure 3(PAR1 37KD, GAPDH36KD)

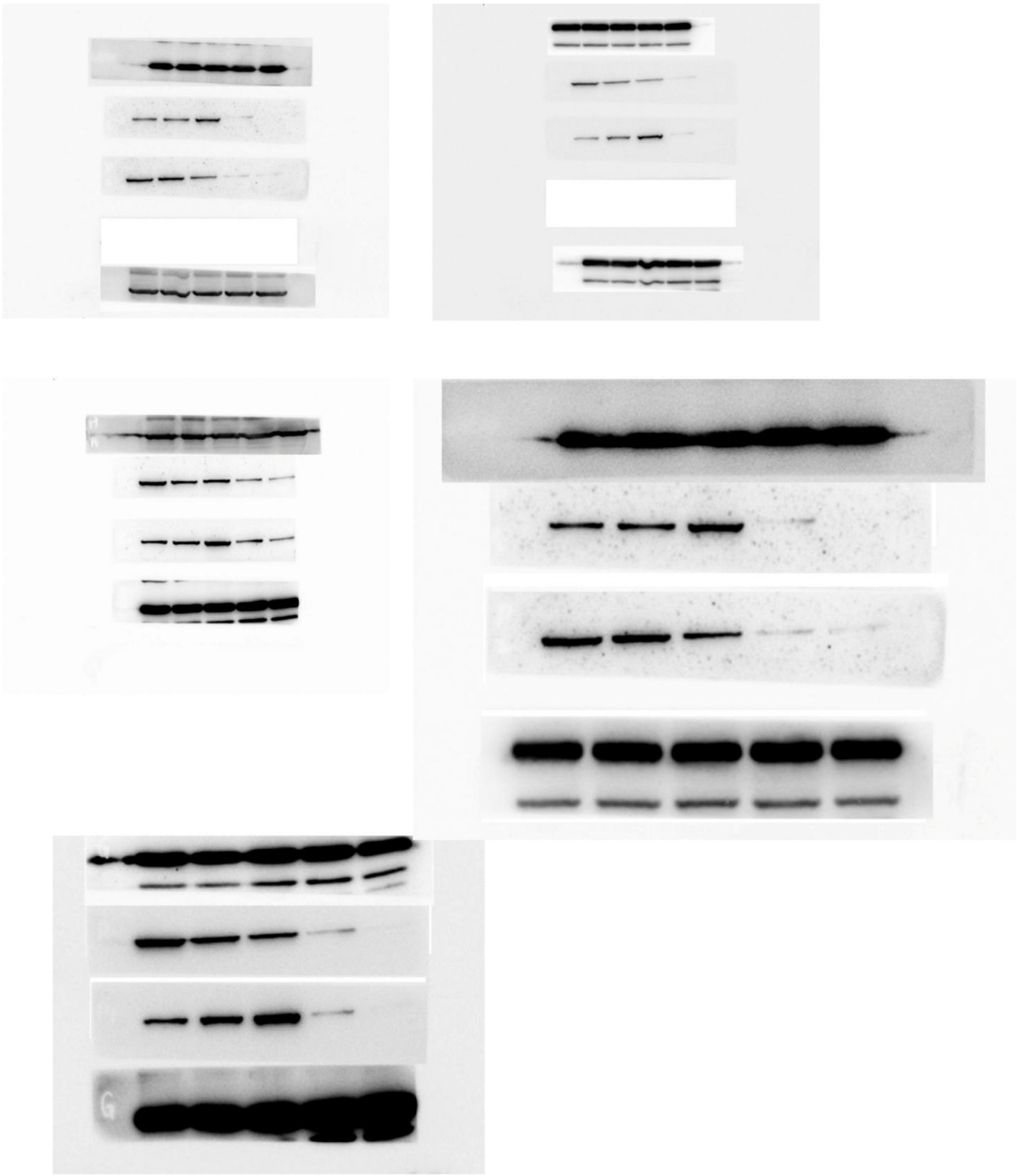

The original figures of Figure 4(PAR1 37KD, GAPDH36KD)

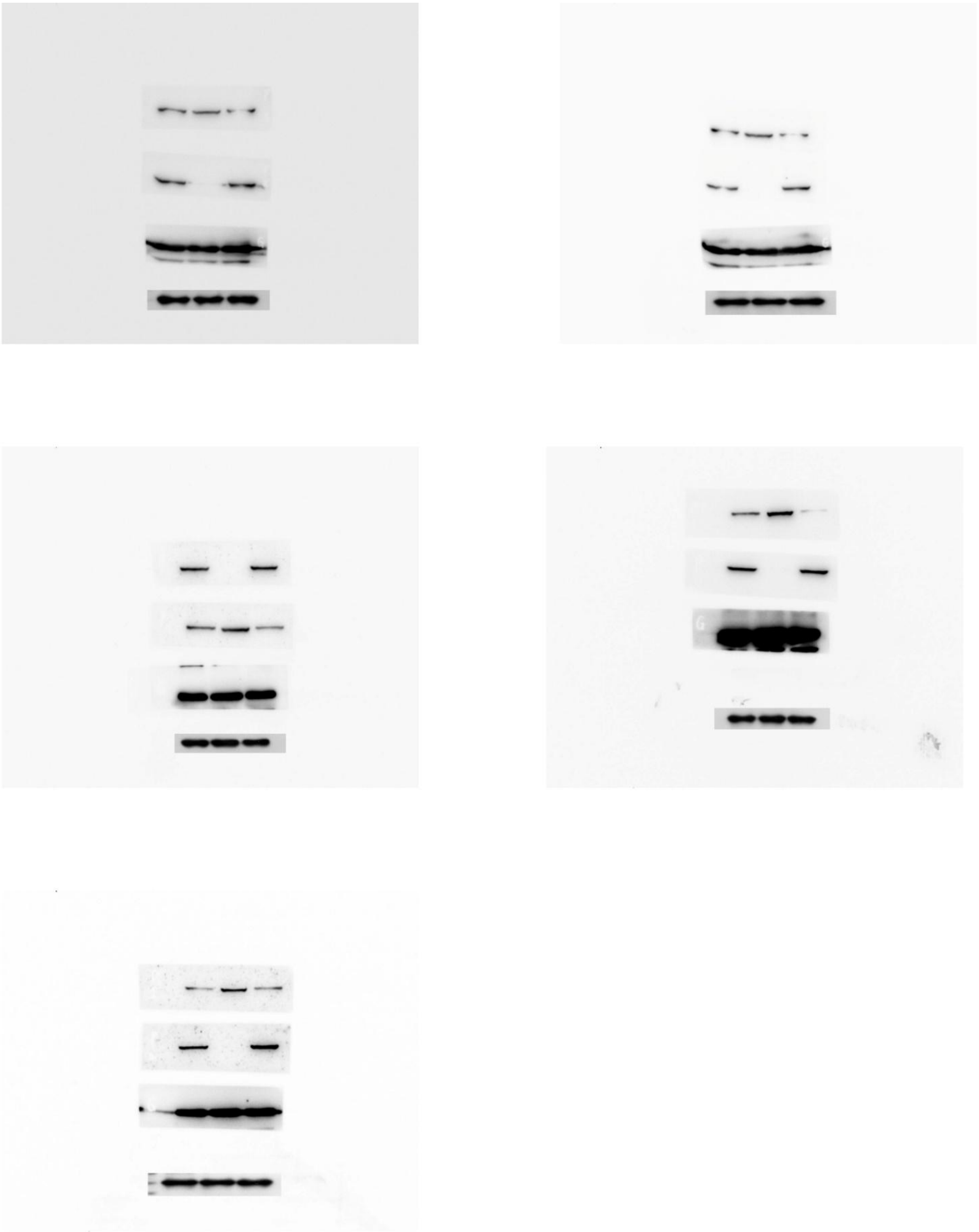

The original figures of figure 5C (PAR1 37KD, GAPDH36KD)

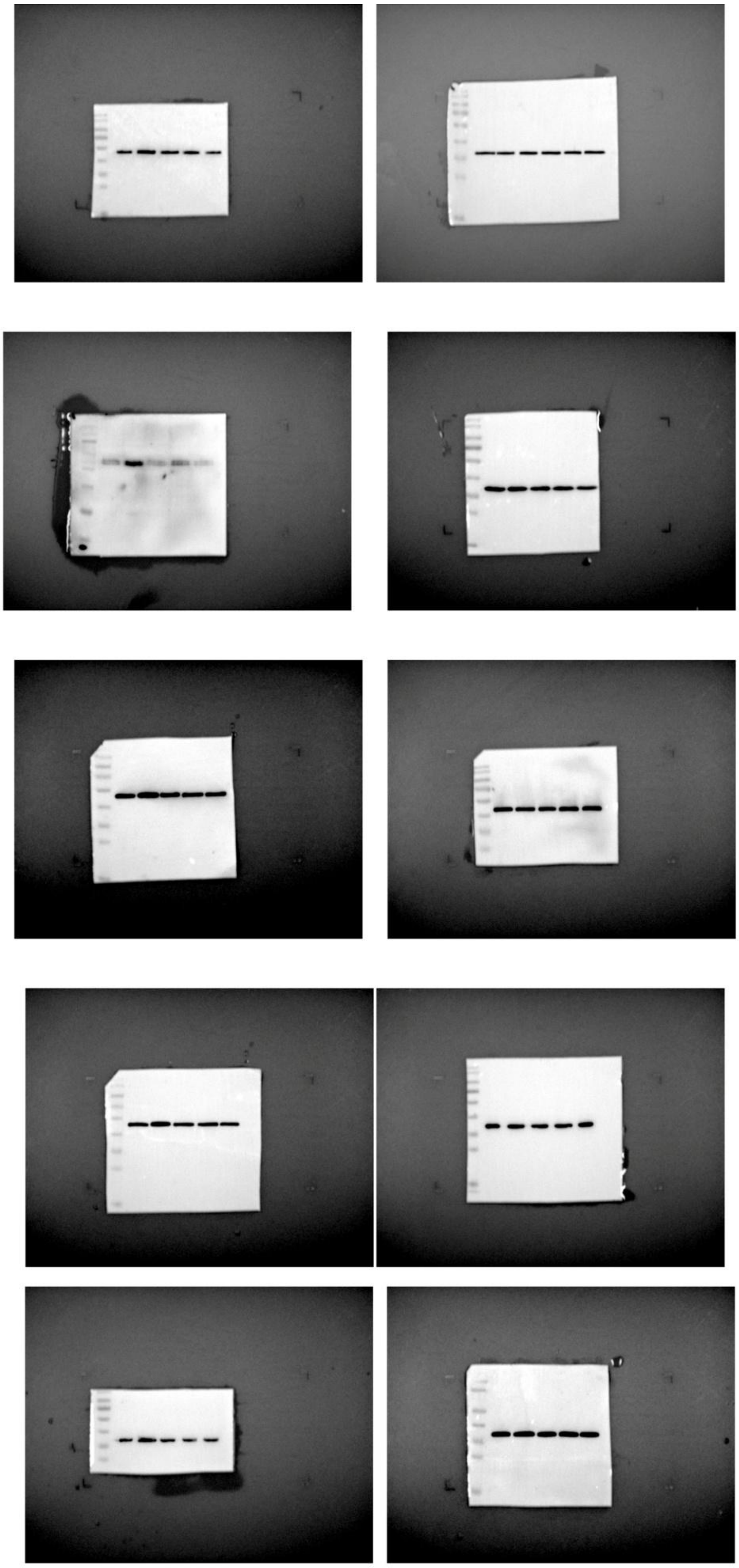

The original figures of Figure 6A (PAR1 37KD, GAPDH36KD)

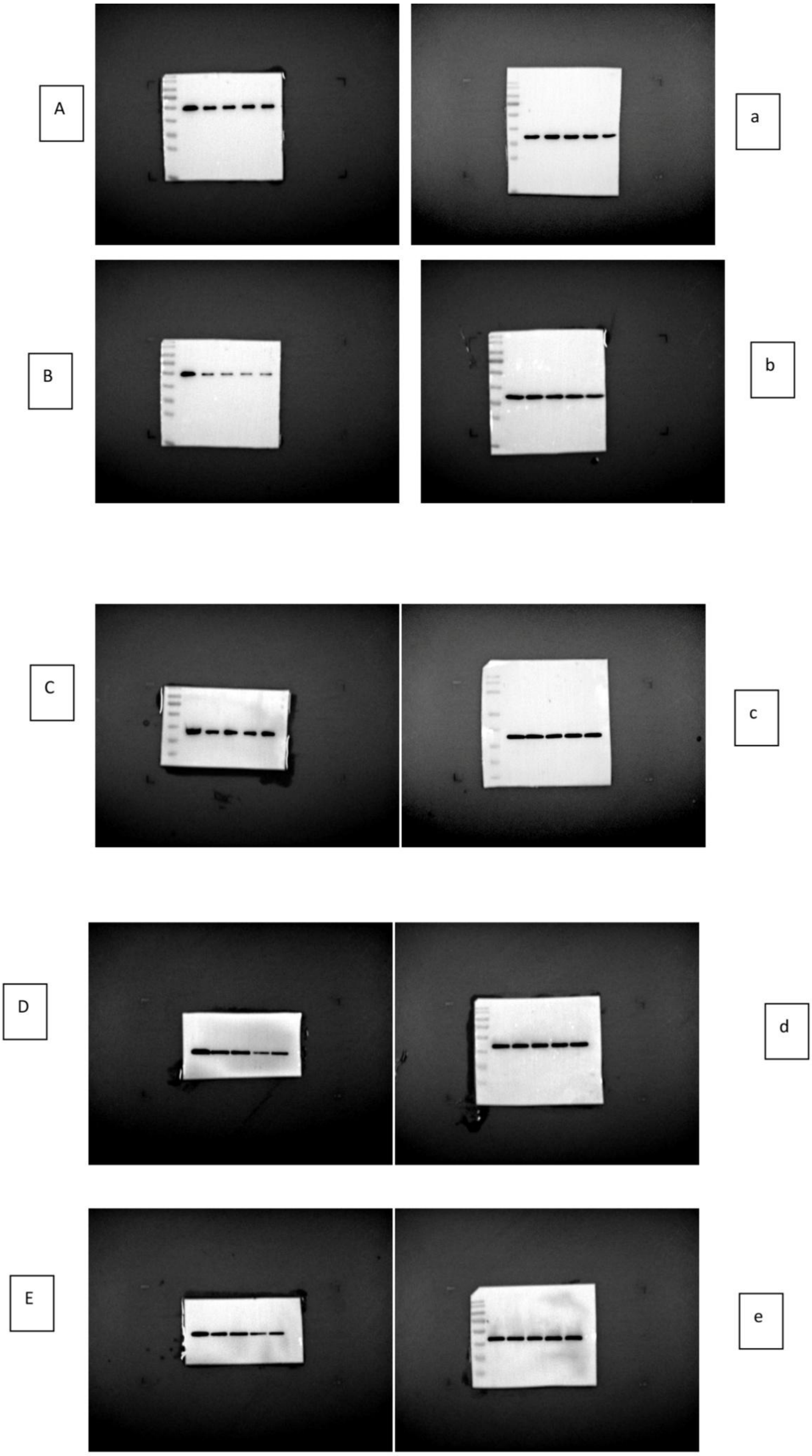

The original figures of Figure 6C (PAR1 37KD, GAPDH36KD)
