# Supplementary material for: TRAP-induced PAR1 expression with its mechanism during AMI in a rat model
Source: BMC Cardiovasc Disord. 2023 Feb 21;23:97. doi: 10.1186/s12872-023-03118-w (PMC9942295; doi:10.1186/s12872-023-03118-w)
Supplement: Supplementary file 3 — Additional file 3. The siRNA sequnces for Rab11A, Rab11B. [file 12872_2023_3118_MOESM3_ESM.pdf]

Supplement 1: The siRNA sequnces for Rab11A, Rab11B。
rat Rab11a (gene ID 81830): sense, 5′- GAGCAGUAG GUGCCUUAU-3′; antisense, 5′-AUAAGGCACCUACUGCUCC-3′;
rat Rab11b (gene ID 79434): sense, 5′-GCAGAUAGCAACAUUGUUA-3′; antisense, 5′-UAACAAUGUUGCUAUCUGC-3′;
rat GAPDH: 5′-GAACGG GAAGCTCACTGG-3′ and 5′-GCCTGCTTCACCACCTTCT-3′. Rat GAPDH was used as an internal control.
